# Age and Gender Recognition Using a Convolutional Neural Network with a Specially Designed Multi-Attention Module through Speech Spectrograms

**DOI:** 10.3390/s21175892

**Published:** 2021-09-01

**Authors:** Anvarjon Tursunov, Mustaqeem, Joon Yeon Choeh, Soonil Kwon

**Affiliations:** 1 Interaction Technology Laboratory, Department of Software, Sejong University, Seoul 05006, Korea; tursunovanvarjon@gmail.com (A.T.); mustaqeemicp@gmail.com (M.); 2 Intelligent Contents Laboratory, Department of Software, Sejong University, Seoul 05006, Korea; zoon@sejong.edu

**Keywords:** human-computer interaction, convolutional neural network, multi-attention module, age and gender recognition, speech signals

## Abstract

Speech signals are being used as a primary input source in human–computer interaction (HCI) to develop several applications, such as automatic speech recognition (ASR), speech emotion recognition (SER), gender, and age recognition. Classifying speakers according to their age and gender is a challenging task in speech processing owing to the disability of the current methods of extracting salient high-level speech features and classification models. To address these problems, we introduce a novel end-to-end age and gender recognition convolutional neural network (CNN) with a specially designed multi-attention module (MAM) from speech signals. Our proposed model uses MAM to extract spatial and temporal salient features from the input data effectively. The MAM mechanism uses a rectangular shape filter as a kernel in convolution layers and comprises two separate time and frequency attention mechanisms. The time attention branch learns to detect temporal cues, whereas the frequency attention module extracts the most relevant features to the target by focusing on the spatial frequency features. The combination of the two extracted spatial and temporal features complements one another and provide high performance in terms of age and gender classification. The proposed age and gender classification system was tested using the Common Voice and locally developed Korean speech recognition datasets. Our suggested model achieved 96%, 73%, and 76% accuracy scores for gender, age, and age-gender classification, respectively, using the Common Voice dataset. The Korean speech recognition dataset results were 97%, 97%, and 90% for gender, age, and age-gender recognition, respectively. The prediction performance of our proposed model, which was obtained in the experiments, demonstrated the superiority and robustness of the tasks regarding age, gender, and age-gender recognition from speech signals.

## 1. Introduction

Human speech is one of the most used sources of communication among mankind. A speech signal consists of information regarding not only the content of speech but also emotions, age, gender, and speaker identity. Moreover, speech signals play an essential role in human–computer interaction (HCI). Nowadays, the speech signal is being used as a primary input source for several applications, such as automatic speech recognition (ASR) [1], speech emotion recognition (SER) [2], gender recognition, and age estimation [3,4]. Additionally, automatically extracting the age, gender, and emotional state of a speaker from speech signals has recently become an emerging field of study. Proper and efficient extraction of a speaker identity from speech signal leads to building applications such as advertisements based on customer age and gender, a caller-agent pairing that appropriately assigns agents depending on the caller identity in call centers. Determining the gender information of a speaker contributes to a performance increase in the speaker recognition system by helping to reduce the search space in the database. Moreover, age recognition helps the systems that are operated using the speaker’s voice command to adapt to the user and provide a more natural human–machine interaction.

Researchers have developed various methods for recognizing age and gender identity from speech signals in the past decade, mainly focusing on the following two issues: determining optimal features and designing a suitable recognition model. In a previous study [5], i-vectors were used as an optimal feature for age estimation, and support vector regression (SVR) was used as an age prediction model. In another study [6], the authors proposed a score-level fusion method for age and gender classification from spectral and prosodic features of speech using the classical Gaussian mixture model (GMM) and support vector machine (SVM) classification models. Another study [3] reports a deep neural network (DNN) architecture used to build x-vectors by mapping a variable-length utterance into a fixed dimensional embedding vector that holds relevant sequential information. The constructed x-vector was then used for age estimation based on the speaker speech signal. A unified DNN architecture to recognize both the height and age of a speaker from short durations of speech was also proposed [7], which improved age estimation by 0.6 years in terms of the root mean square error (RMSE) over the classical SVR. The authors of [8] proposed a novel age estimation system based on Long short-term memory (LSTM) recurrent neural networks (RNN) that can deal with short utterances using acoustic features. Another notable method was proposed, which utilized a convolutional recurrent neural network (CRNN) for age and gender prediction from speech utterances [4].

Although researchers have suggested several methods for age and gender recognition from speech signals, extracting optimal feature sets and designing high-performance classification models remains challenging. One of the reasons for low results in age classification when using acoustic features is the similarity of frequency-related acoustic features across different age groups [9,10]. The proposed i-vectors and x-vectors uses acoustic features to form embedding vectors. The i-vectors and x-vectors cannot provide high classification results, especially for age classification, using DNN, SVM, and GMM even though they are more robust compared to acoustic features. It is difficult for both training and achieving high performance using traditional machine learning algorithms such as SVM and GMM when the size of the input features is big. State-of-the-art results have been achieved by properly designing a convolutional neural network (CNN) to solve challenging tasks such as ASR [11], audio classification [12], and many more other speech-processing-related tasks. Building the suitable architecture of the CNN model is hard and challenging to achieve high performance. Hence, researchers have been continuously attempting to achieve better results in speech-processing-related tasks by utilizing the power of deep learning algorithms.

In this study, we proposed CNN with a specifically designed multi-attention module (MAM) that addressed the two main aforementioned issues of age and gender classification using speech signals. The extraction of salient high-level features, which is the first issue, is addressed by designing MAM to efficiently manage spatial and temporal salient features from the input data. Our proposed MAM mechanism uses a rectangular shape filter as a kernel in convolution layers and consists of two separate time and frequency attention mechanisms. The time-attention branch learns to detect the temporal cues of the input data. In contrast, the frequency attention module extracts the most relevant features to the target by focusing on the spatial features on the frequency axes. Both extracted features are then combined to complement one another and build more robust features for processing in the subsequent layers. We created a feature learning block (FLB) consisting of convolution, pooling, and batch normalization layers to extract local and global high-level features. We achieved the best recognition score for age and gender classification using the proper combination of FLB, MAM, and the fully connected network (FCN) with the SoftMax classifier, which is the neural network activation function that converts the final output values of the network into a vector of probabilities. We tested our proposed model over the Common Voice [13] and Korean speech recognition datasets to experimentally prove the efficiency and robustness of the proposed model. Originally, both datasets were developed for the ASR task, but they can also be used for age and gender recognition. The Common Voice dataset is a multilingual collection of transcribed speech. However, the Korean speech recognition dataset was only in the Korean language. The results of the proposed framework are presented in Section 4. The primary contributions of our proposed system are as follows:

- We propose a CNN model with a MAM to adequately capture spatial and temporal cues from speech spectrograms, which ensures high performance and robustness for age and gender recognition.
- A specially designed MAM mechanism that consists of separate time and frequency attention modules was proposed to learn and select the most relevant features to the target from the input data using a rectangular shape filter as a kernel in the convolution layers to provide and enhance the performance of the baseline age and gender recognition CNN models.
- We developed and tested three different CNN models for age, gender, and age-gender classification problems to analyze and represent the effectiveness of the MAM module when it was placed in various parts of the CNN model. We experimentally proved the importance of placing the MAM mechanism in different parts of the CNN models.
- Our proposed age and gender classification system was evaluated using the Common Voice and locally developed Korean speech recognition datasets. Our suggested model achieved 96%, 73%, and 76% accuracy scores for gender, age, and age-gender classification, respectively, using the Common Voice dataset. The Korean speech recognition dataset results were 97%, 97%, and 90% for gender, age, and age-gender recognition, respectively. Our proposed model achieved the best recognition results in all three classification tasks when MAM was placed between two FLBs compared to the various classification methods. The experiments showed the importance of the location of MAM in the CNN model, and the analysis results are provided in the experimental results section.

The remainder of this manuscript is structured as follows: the literature review regarding age and gender classification is provided in Section 2, and a detailed explanation of the proposed system and its primary components, datasets, experimental setup, and evaluation metrics are described in Section 3. The experimental results are presented in Section 4. Discussion and comparative analysis are provided in Section 5. Finally, the conclusions and future directions are presented in Section 6.

## 2. Literature Review

There are several applications in the digital audio signal processing domain, such as speaker identification [14] and recognition [15], speaker segmentation, and personalizing human–machine interactions, that have challenges in automatically identifying gender and age from voice and speech signals [16]. Researchers can now measure the properties of speech signals, both temporal and frequency related important cues, by recent developments and advancements in voice recording technology [2]. Gender and age recognition and identification from speech signals is challenging in this domain, which should be automated or smart for determining the speaker gender from voice signals. The classification of gender and age from direct speech signals can be logically related to the time or frequency domains. In the time domain analysis, we directly measure the speech signals considering the content of a signal for evaluating information regarding a speaker; on the other hand, in the frequency domain analysis, the frequency content of a speech signal is used to form a spectrum for evaluating information regarding a speaker, which is analyzed accordingly [17,18]. The smart gender-age recognition system used the main variation in the levels of power and the frequency content of the two genders to identify the gender-based speech signals [9,10]. In this regard, Ali et al. [19] suggested a technique for encoding speech signals in the digital jurisdiction using principal component analysis (PCM) and then determined the signal frequency information using deep frequency transformation (DFT) [20]. The speech signal, on the other hand, is entirely composed of real points, such as frequency, amplitude, pitch, and time. Hence, the proposed method employs real point DFT to improve the efficiency and effectively identify the gender and age of speakers through speech. The proposed method obtained an identification rate above 80% by utilizing the short-time Fourier analysis (STFA).

A system for determining gender based on speech signals was recently proposed [15] that utilized a fast Fourier transform (FFT) with a 30 ms Hamming window and a 20 ms overlap for signal analysis. Each 10 ms spectrum was further filtered to adhere to the Mel Scale, yielding a vector of 20 spectral coefficients. The mean and variance of the Multilayer Feature Switch Card (MFSC) vectors were determined in each window, and 40 coefficients were concatenated to generate a feature vector. The feature vector is then normalized to ensure that the classifier captures the relationship between the frequency of the spectrum rather than the individual frequencies. The authors used a neural network such as MLP, DNN, CNN, and LSTM as a classifier in this method and obtained an accuracy rate above 90%. Similarly, Martin A. F. et al. [19,21] provided one of the most innovative revelations in the field of voice or speech signal-based gender and age identification. The study suggested multiple linguistic detections for multiple speakers. Rather than focusing on a single speaker, multi-speaker analysis was used in this study to address the practical environment with higher efficiency and to obtain a reasonable recognition rate. Khan et al. [22] suggested a fuzzy logic-based gender classification structure that was trained using several metrics, such as the power amplitude, total harmonic distortion, and power spectrum. Although the study yielded positive results, as the rule base grows when utilizing the fuzzy logic, the scheme becomes more complex and lacks the added benefit of learning owing to these issues; the proposed method failed to achieve a high accuracy [23]. Nowadays, researchers have used deep learning approaches to recognize emotions [2,24,25,26] in speech and human action in the frame of sequence [27].

In this advance era, researchers have proposed hybrid methods [28,29] and systems to address the limitations of the age and gender recognition domain, which appear to offer a potential answer to the problem; however, they also significantly increase the system complexity, and the problem of noise has not been considered. Furthermore, owing to higher complexity, these systems require more computational time [23,30], which is addressed in this study. Furthermore, Prabha et al. [31] created a system for classifying gender based on energy; the signal transformation was performed using FFT, and the system secured a 93.5% efficiency. Several methods use machine learning techniques for classification [32,33] and classify gender and age with high accuracy of 92.5%. The proposed system utilizes a windowing and pre-emphasis technique for noise cancellation during the preprocessing phase. In gender-age recognition, an x-vector (fixed-length representations of variable-length speech segments) framework was recently proposed as a replacement for i-vectors (the segment is represented by a low-dimensional) [34]. For age and gender prediction, Ghahremani et al. presented an end-to-end DNN employing x-vectors [3]. Hence, the main difficulties in i-vector/x-vector approaches for obtaining high-performance outcomes are the intensive acquirement of a large amount of data and the complex architecture to train the system. This research investigated voice signals and suggested a better, smarter AI-based age-gender classification system. Our system uses an end-to-end AI-based approach with a lightweight attention mechanism to selectively focus on important information and efficiently recognize the age and gender of speakers.

## 3. Proposed Age and Gender Classification Methodology

**Figure 1:**
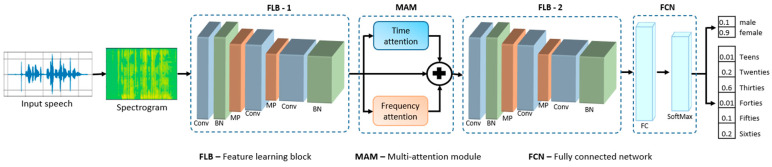
An overview of the proposed age and gender recognition framework. FLB-1—first FLB, which extracts local feature maps. MAM selects the unique characteristics of both time and frequency. FLB-2—second FLB that learns high-level salient feature maps. FCN extracts global features and performs classification. Conv—convolution layer, BN—batch normalization layer, MP—max-pooling layer, FC—fully connected layer.

In this section, we explain the proposed framework and its related MAM module. Our suggested framework utilizes a MAM for the recognition of both the age and gender of a speaker from speech spectrograms. The proposed age and gender recognition CNN model is shown in Figure 1. The speech spectrogram contains significant information regarding speaker age, gender, speaking style, emotional content, etc. One of the reasons for the importance of the speech spectrograms is that a human also processes the sounds in the form of different frequencies over time in the ear [35]. Additionally, a two-dimensional representation of the speech signal is a suitable input data for CNN models for speech analysis. The proposed model architecture consists of two feature learning blocks (FLB)s, a specially designed MAM, and a fully connected network (FCN), which recognizes the age and gender from the spectrogram generated from the speech signal. A detailed explanation of the spectrogram generation and the proposed framework components are presented in the subsequent sections.

### 3.1. Spectrogram Generation

A spectrogram is one of the most used visual input representations of speech signals in speech analysis tasks, such as ASR [36] and SER [24] using deep learning (DL) models. It demonstrates the signal strength over time at different frequencies present in a particular waveform. In the spectrograms, time is shown on the horizontal axis, while the vertical axis represents the frequency, and the yellow color of each point in the graph corresponds to an amplitude of a certain frequency at a particular time.

**Figure 2:**
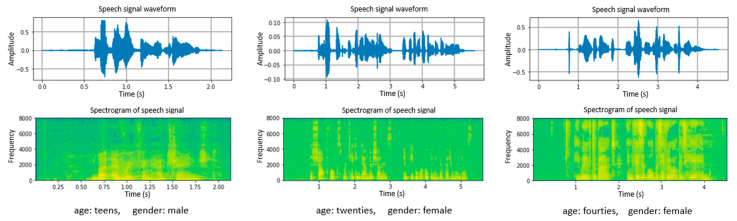
Audio waveforms and corresponding spectrograms of speech samples.

The Short-time Fourier transform (STFT) is applied to a discrete signal to generate a spectrogram of the speech signal. The speech signal is divided into short overlapping segments of equal length, and then a fast Fourier transform (FFT) is applied to each frame to compute its spectrum. For a given spectrogram *S*, the strength of a given frequency attribute *f* at a given time *t* is represented by the darkness or color of the corresponding point *S(t, f)*. We extracted speech spectrograms, as shown in Figure 2, using STFT for each speech signal in the dataset. Figure 2 presents the audio waveforms and generated corresponding spectrograms of the speech samples.

### 3.2. Feature Learning Block (FLB) Explanation and Configuration

Current state-of-the-art results in the field of computer vision are achieved by utilizing the power of CNN for different tasks, such as image classification [37], object detection [38], and action recognition [39]. CNN models have demonstrated their superiority not only in the field of computer vision tasks but also in ASR [40] and SER [33]. The performance of CNN models inspired us to utilize their efficiency for age and gender classification using speech signals. Generally, a CNN model consists of the following three main components: convolution layers, pooling layers, and fully connected layers. Convolution layers are formed by specifying the number of filters and kernel size, which define the receptive field and stride size, and is responsible for the amount of movement over the input. The primary function of the convolution layer is to learn and extract high-level features from the input data. The output of the convolution layer is fed to pooling to decrease processing time by reducing the dimensionality of the feature maps. These layers are usually arranged in the form of a hierarchy where we can use any number of convolution layers followed by pooling layers to build robust and salient feature maps.

Our FLB was formed by utilizing three convolutions (C), two max-pooling layers, and two batch normalization (BN) layers. Details regarding the configuration of our proposed model layers, input tensor, which is a high dimensional vector, output tensor, and parameters are provided in Table 1. We used 120 kernels with a size of 9 × 9 and a stride of 2 × 2 with a padding configuration in the first convolution (C1) layer to extract useful local features from the input spectrogram. In addition, a rectified linear unit (ReLU) was used as an activation function in all the convolution layers to achieve better performance and to generalize the model during the training. The second convolution (C2) layer includes 256 filters of size 5 × 5 and a stride of 1 × 1 with the same padding setting to generate a feature map to the next layer for further processing. The third convolution (C3) layer has 384 kernels of size 3 × 3 with the same stride and padding configuration of C2 to extract deeply hidden cues from the input data. The MP layer with a pool size and a stride of 2 × 2 is applied after the BN layer in C1, which is followed by C2. The MP layer reduces both the dimensions of the feature map and the network computation cost. The BN layer was placed after C1 and C3 to normalize and rescale the feature map of the speech spectrogram. Our FLB uses quadratic-shaped filters to learn the relationship between the time and frequencies of the speech signal and extracts high-level salient feature maps.

### 3.3. Proposed Multi-Attention Module

**Figure 3:**
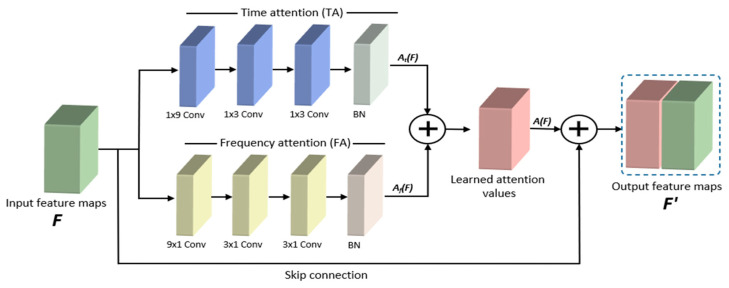
A detailed overview of the MAM module structure. Input feature maps fed to time and frequency attention modules to capture the critical parts of both time and frequency features. Learned attention values are concatenated with input feature maps using a skip connection to produce output feature maps.

The utilization of attention mechanisms in deep learning models has demonstrated its strength in model performance and robustness in solving several different tasks, such as machine translation [41], text classification [42], sound event detection [43], and speech emotion recognition [33]. The working mechanism of attention is to select essential features to the target by focusing on the extracted features. Not all extracted features are equally relevant to the target in any deep learning task. Feature maps generated using CNN from speech spectrograms contain information regarding particular regions of the input data. However, it is challenging to capture temporal cues using CNNs. As the spectrogram of the speech signal represents temporal and spatial information, we must capture all the valuable features of the input data to achieve high performance. To solve this issue and improve the performance and robustness of the classification model, we designed a new attention module using two-stream, time, and frequency attention mechanisms separately, which have various properties. The detailed architecture of the MAM is shown in Figure 3.

From the given input feature map F ϵ *R^H×W×C^*, a learned 3D attention map A(F) ϵ *R^H×W×C/r^* is first computed, where *r* is the reduction ratio, and the final refined feature map is calculated as follows:

(1) $$F^{\prime }=F+A\left(F\right)$$

We applied a residual learning scheme along with the MAM to facilitate gradient flow. To compose an efficient and robust attention module, we first compute the time attention A_t_(F) ϵ *R^H×W×C/2r^* followed by the frequency attention A_f_(F) ϵ *R^H×W×C/2r^* values at two separate branches. The final 3D attention map is computed as follows:

A(F) = A_t_(F) + A_f_(F) (2)

The time attention module exploits the relationship of the particular frequency with the time axis and contains specific frequency-related features at different times. To achieve this, we applied three convolution operations with various rectangular shape filters followed by batch normalization (BN) to the input feature maps F ϵ *R^H×W×C^*. The kernel size of the first convolution layer is set to 1 × 9, and the input channels are reduced with the reduction ratio *r*. The next two convolution layers have a 1 × 3 kernel size and the same input channels. We used BN to normalize and rescale the computed time-attention map. The final time attention map is calculated as follows:

(3) $$A_{t}(F)=BN(J_{3}^{1\times 3}(J_{2}^{1\times 3}\left(J_{1}^{1\times 9}(F\right)))$$

where J denotes a convolution operation, BN denotes a batch normalization operation, and the superscripts denote the convolution filter sizes.

The frequency attention module learns the importance of each frequency to the target during training. The frequency attention module has the same number of convolution operations and BN; however, it has different kernel sizes. Three convolution operations are applied to the input feature maps of F ϵ *R^H×W×C^* with kernel sizes of 9 × 1, 3 × 1, and 3 × 1, respectively. The output dimension of this attention module is the same as that of the time-attention module. The final frequency attention map is computed as follows:

(4) $$A_{f}(F)=BNJ_{3}^{3\times 1}(J_{2}^{3\times 1}\left(J_{1}^{9\times 1}(F\right)))$$

where J denotes a convolution operation, BN denotes a batch normalization operation, and the superscripts denote the convolution filter sizes.

After computing the attention maps from two separate branches, we obtain two different attention values that focus on the most time-related and frequency-related features of the input feature maps. To build our final 3D attention map A(F), we combined these two attention maps (Equation (2)) because the attention values computed at two different branches complement one another owing to the fact that the input feature map is the same for both branches, while the extracted attention maps are different. We concatenated the input feature map F and attention weights obtained from MAM A (F) to build a more informative feature map to feed for further processing in the second FLB.

### 3.4. Age and Gender Classification Datasets

We used Common Voice [13] and Korean speech recognition [44] datasets to evaluate and demonstrate the proposed model significance and robustness. Both datasets were originally created for developing a speech recognition system. However, they can also be used for conducting research and developing age and gender recognition systems. A detailed description of the datasets is provided in the following subsections.

#### 3.4.1. Description of the Common Voice Dataset

The primary purpose of the Common Voice dataset [13] is to accelerate the research domain of automatic speech recognition technologies. However, it can be used to research and develop other speech domain tasks, such as language identification, speaker age, and gender recognition. It consists of a collection of massively multilingual transcribed speech that is publicly available. To obtain reproducible results, we used the English subset of the dataset, which dominated the other languages in terms of the number of speakers and validated files; this was available through the Kaggle website [45]. This dataset was divided into valid and invalid subsets. The valid subset contains audio files that at least two people validated as the audio matches the text. Each subset is separated into training, development, and test groups on the Kaggle website. All audio files were labelled for the speech recognition task, however, not for age and gender classification. Three annotators labelled the audio files, and the final labels were assigned based on the majority voting method. Thus, we selected audio files based on the age and gender information from the dataset. Detailed information regarding the age groups, gender, and the number of files in the dataset is provided in Table 2. Our final selected dataset includes both genders along with six age groups named teens, twenties, thirties, forties, fifties, and sixties. The speakers in the training, development, and test sets were significantly different from one another.

#### 3.4.2. Description of the Korean Speech Recognition Dataset

The Korean speech recognition database was developed using 24,000 sentences related to utilizing the sentence for AI-based systems, involving 7601 Korean speakers collected from residents of seven regions within the province of Korea. This study was financially supported by the AI database project of the National Information Society Agency (NIA) in 2020. The recordings occurred in a silent room using a personal smart device. The participants were requested to record themselves according to the prepared sentences. After each sentence was recorded, they listened to and checked the quality of the voice data. If not correctly recorded, the same sentence was re-recorded until the recording quality was sufficient. The evaluation of the recorded data was conducted by professional researchers. The database contains records of three situations, AI secretary, AI robot, and Kiosk, from the following three groups: children, adults, and the elderly. The total recording time was 10,000 h. We selected random samples from the AI secretary situation for our experiments and evaluated our model. A detailed description of the dataset is provided in Table 3.

### 3.5. Experimental Setup

We implemented our proposed model using TensorFlow [46], an open-source platform for developing machine learning algorithms, and the Python [47] programming language. Speech spectrograms were generated using the librosa [48] Python library for audio and music analysis. The Common Voice dataset consists of the following three parts: training, development, and test sets. The training part was used to train our proposed model, and the development part was used to evaluate our trained model while conducting the experiments. The test set was only used to verify the model performance after training was complete. We divided the Korean speech recognition dataset into three subsets, with 70% utilized for training, 15% for development, and the remaining 15% for testing the model performance. The proposed model was trained with 50 epochs using the Adam optimizer with a learning rate of 0.0001. We conducted experiments with various batch sizes, and the best result was achieved with a batch size of 256 samples. To maintain a balance between the computation cost and model performance, input spectrograms, with a size of 64 × 64 pixels, were chosen as the optimal option among the other 32 × 32 and 128 × 128 pixels. Two NVIDIA GeForce RTX 3080 GPUs with ten GB of onboard memory were used to conduct all the experiments.

### 3.6. Evaluation of Model Testing Performance

To measure the proposed model performance for age and gender classification, we utilized the statistical parameters computed from the model prediction and ground truth. Definition of the terms used to calculate evaluation metrics is given in Table 4. The number of positively predicted samples correctly matching with the actual positive labels is called the true positive (TP) value, whereas the false negative (FN) value is the number of positive predicted samples that do not match with the positive ground truth labels. True negative (TN) indicates the correctly predicted negative samples with actual values, and false positive (FP) indicates the samples that are predicted as negative, but the actual labels are positive samples.

The statistical parameters assist in computing other factors that are used to evaluate the model performance and robustness. The accuracy score is calculated using Equation (5), and it indicates how many of the classes were correctly predicted. Utilizing only the accuracy factor is insufficient for measuring the model performance and effectiveness. Thus, additional measurement factors, such as recall (Equation (6), precision (Equation (7), F1-score (Equation (8), weighted accuracy, and unweighted accuracy, are required to measure the proposed model efficiently.

(5) $$\mathrm{Accuracy}=\frac{\mathrm{TP}+\mathrm{TN}}{\mathrm{TP}+\mathrm{TN}+\mathrm{FP}+\mathrm{FN}}$$

(6) $$\mathrm{Recall}\;=\frac{\mathrm{TP}}{\mathrm{TP}+\mathrm{FN}}$$

(7) $$\mathrm{Precision}=\frac{\mathrm{TP}}{\mathrm{TP}+\mathrm{FP}}$$

(8) $$F1-\mathrm{score}\;=\frac{2\times \mathrm{TP}}{2\times \mathrm{TN}+\mathrm{FP}+\mathrm{FN}}$$

## 4. Experimental Results

We empirically evaluated our proposed classification framework using the Common Voice dataset [13] and a Korean speech recognition dataset to demonstrate the model significance and robustness. We checked the performance of our system and compared it with other CNN model architectures and methods with the same tasks. We conducted experiments to classify the three different tasks. The first task was gender classification. The proposed model was trained to classify the speaker into male and female classes using speech spectrograms. In the second task, the model was trained only for age classification without predicting gender information. The final task involved the recognition of both age and gender from the speech spectrogram. Moreover, five different CNN models were developed and evaluated for all the classification problems. The prediction performances of all five CNN models are shown in Table 5.

The first CNN model (model-1 in Table 5) consists of two FLBs, which are placed one after the other, and the FCN without MAM. This model was used as the baseline model for analyzing the effectiveness of other CNN models with attention modules. We used two FLBs in order to keep the balance between model complexity and performance as well as to avoid model overfitting problems. The second model (model-2 in Table 5) was built using two consecutive FLBs and a time attention module (TAM), followed by FCN. This model was developed to verify the effectiveness of the TAM mechanism. The third model (model-3 in Table 5) has the same architecture as the second model except that TAM was replaced with frequency attention module (FAM). For the analysis of whether TAM and FAM feature complement each other when combined or not, we developed the CNN model (model-4 in Table 5) using two consecutive FLBs and multi-attention module (MAM), followed by FCN. The last model (model-5 in Table 5) architecture consisted of FLB, MAM, FLB, and FCN in sequential order. The primary purpose of the last model is to analyze and represent the effectiveness of the MAM module when it is placed in various parts of the CNN model.

Table 5 presents the evaluation results of the five CNN models for the Common Voice and the Korean speech recognition datasets. The output from the model is the class probabilities taken from the SoftMax layer. The class with the highest prediction probability was taken as the final model prediction class. Then accuracy score was calculated using the final predicted class and original labels. Speech spectrograms were utilized as the input features for all the models in the classification tasks. All developed CNN models with attention modules achieved higher classification accuracy for both databases over the baseline CNN model in all three classification tasks. Model-3 achieved a better classification accuracy over model-1 and model-2. It means that FAM features are more effective comparing to TAM features as well as it increased classification accuracy considerable over model-1. The results of model-4 indicating the efficiency of a specifically designed MAM module for age and gender classification using speech spectrograms. The highest performance was achieved using the proposed model-5 among the other CNN models, which indicates that the location of the MAM module in the CNN models is significant for obtaining high accuracy.

Table 6 presents the statistical parameters obtained from the model prediction of the test data to recognize speaker age and gender information from the speech spectrograms. The age and gender classification for the Common Voice dataset is twelve classes classification problem. The age range for each class was ten years. However, the age range in the Korean speech recognition dataset was different for each class. The age range of the children group was between three to 10 years, while that of the adult group was between 20 to 59 years. The 60 to 79 age range was considered as the older group.

The highest precision, recall, and F1-score values obtained were 89%, 89%, and 88%, respectively, for the Common Voice dataset. For the Korean speech recognition dataset, these values were 99%, 99%, and 92%, respectively. The results clearly indicate that the recognition rate is higher when the age range difference increases. The average recognition accuracy obtained was 75% and 90% for the Common Voice and Korean speech recognition datasets, respectively.

**Figure 4:**
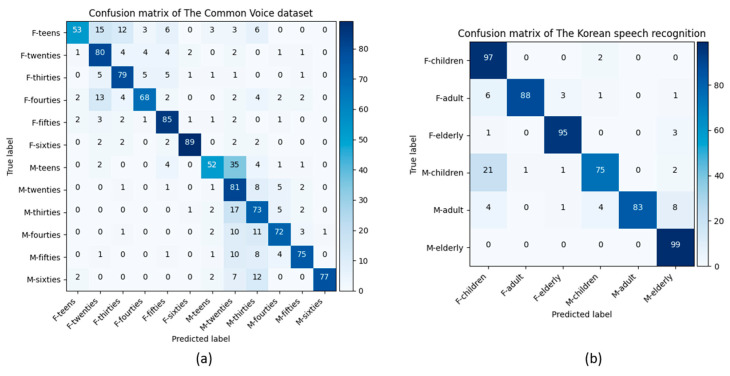
Obtained confusion matrices of the proposed model for the Common Voice (**a**) and the Korean speech recognition (**b**) dataset in age and gender classification with 75% and 90% average recognition rates. F and M denote the female and male genders, respectively.

Figure 4 presents the confusion matrices obtained from the model predictions and the test data labels for the age and gender classification problem for the Common Voice and Korean speech recognition datasets. The best class-wise accuracy for the Common Voice dataset was achieved in the F-sixties (89%) and M-twenties (81%). However, the accuracy rates of both the F-teens and M-teens classes were 53% and 52%, respectively. The proposed model confused the predictions of F-teens and M-teens with F-twenties and M-twenties, respectively. One of the main reasons for this confusion is the similarity of the acoustic features such as pitch, the fundamental frequency, and formants of the F-teens and M-teens with F-twenties and M-twenties age groups [9,10]. As our proposed model captures information from the frequency and time of the generated speech spectrograms, the frequency features of the male and female teens group are mostly similar to those of the twenties group. This similarity feature [49] between the female and male gender groups was also observed in the Korean speech recognition dataset. Most M-children were confused with the F-children class in the model prediction. The voices of children are significantly similar for males and females in terms of acoustic features. The highest classification accuracy for the Korean speech recognition dataset was obtained for F-children and M-elderly with a 97% and 99% accuracy, respectively.

Table 7 presents the precision, recall, F1-score, and weighted and unweighted accuracy results obtained from the model prediction for age classification using the test data for the Common Voice and the Korean speech recognition datasets. The highest precision, recall, and F1-score values were 87%, 83%, and 81%, respectively, for the Common Voice dataset. The values for these parameters were 98%, 99%, and 97% for the Korean speech recognition dataset. When male and female teens were combined into one group, it affected the overall recognition accuracy in the Common Voice dataset. However, the average recognition accuracy for the Korean speech recognition dataset increased in the age classification task. As shown in Figure 4b, most of the confusion occurred for the M-children with the F-children class. When these two classes were combined, the confusion between the M-children and F-children groups were resolved; as a result, the overall classification accuracy increased. The average recognition accuracy obtained was 72% and 96% for the Common Voice and Korean speech recognition datasets.

**Figure 5:**
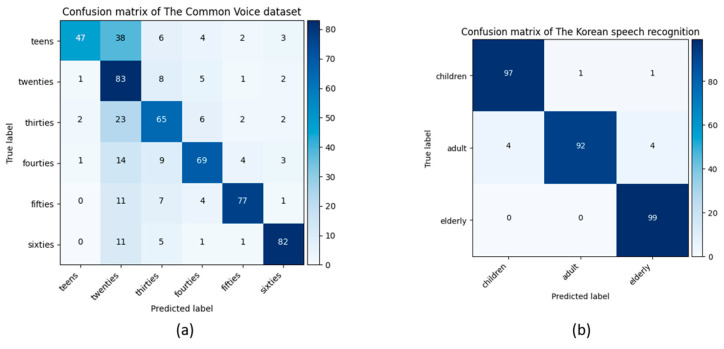
Obtained confusion matrices of the proposed model for the Common Voice (**a**) and Korean speech recognition (**b**) datasets for age classification with 72% and 96% average recognition rates.

Figure 5 illustrates the confusion matrices for age classification from the speech spectrograms obtained from the model predictions and the actual labels for the Common Voice (a) and the Korean speech recognition (b) datasets. The best accuracy among the age classes for the Common Voice dataset was achieved in the twenties age range (83%), while the lowest accuracy was observed in the teens class. The proposed model mostly confused the predictions of the teens with the twenties group owing to the similarity of the frequency features. The highest classification accuracy for the Korean speech recognition dataset was obtained for the elderly group (99%). The age difference between the age groups in the Korean speech recognition dataset was noticeably different. Hence, their frequency features were also different [49]. Our proposed model can capture these different features during the model prediction. As a result, each age group had an accuracy score above 90%.

Table 8 presents the statistical factors obtained from the model prediction for gender classification using the test data for the Common Voice and the Korean speech recognition datasets. The highest precision, recall, and F1-score values achieved the same 97% in the male class for the Common Voice dataset. The values for these parameters were 96%, 97%, and 96% for the Korean speech recognition dataset. The average recognition accuracy of 96% was obtained for both the Common Voice and Korean speech recognition datasets.

**Figure 6:**
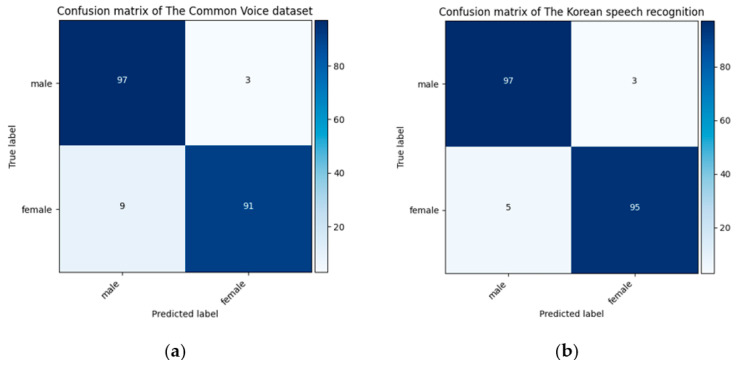
Obtained confusion matrices of the proposed model for the Common Voice (**a**) and Korean speech recognition (**b**) dataset for gender classification with average recognition rates of 96% for both datasets.

The confusion matrices for gender classification from the speech spectrograms are presented in Figure 6. The results were obtained from the model predictions and the actual labels for the Common Voice (a) and Korean speech recognition (b) datasets. The best accuracy of 97% was achieved for gender classification in the male class for both datasets. Over 90% classification accuracy was achieved for each gender class on both datasets, even though the Common Voice dataset is gender imbalanced. This indicates that our proposed model capable of learning differential features from the imbalanced dataset and demonstrated its superiority to all three classification problems.

## 5. Discussion and Comparative Analysis

In this study, we proposed a CNN model with a specifically designed MAM for age and gender classification using spectrograms generated from the speech signal. The MAM was designed to capture the most relevant salient features from both the time and frequency axes of the input spectrogram. MAM is considered to be our primary contribution to age and gender classification from speech signals. Additionally, we built an FLB scheme to extract high-level salient feature maps from the input data. To the best of our knowledge, this is the first CNN model with a MAM mechanism proposed for age and gender classification using speech spectrograms. We thoroughly investigated the literature regarding age and gender recognition from speech signals and found two main issues, the first of which is the difficulty capturing the essential features for age classification because most of the acoustic features relevant to the speech frequency are similar in the age groups of children, teens, and twenties [9,10,49]. Several researchers have attempted to develop various techniques in this domain by utilizing hand-crafted features using classical machine-learning classification models [5]. However, the recognition score remained low. The second issue is the design of a proper classification model [6,50]. Recently, deep learning models have been applied for age and gender recognition [7]; however, the aforementioned issues remain unresolved.

In this study, we addressed these issues and designed a novel CNN model with a MAM mechanism. MAM consists of two separate branches responsible for learning and detecting essential features that are mostly related to the target in both the frequency and time domains. We used a rectangular shape filter as a kernel on the convolution layer of the time attention and frequency attention branches to manage the most relevant features. A proper combination of FLB and MAM captures both local and global high-level salient features in our proposed model. An FCN with a SoftMax classifier was used for our proposed model. A speech spectrogram, which is a 2D visual representation of frequencies over time, was used as an input to our proposed model. Table 1 lists the detailed configuration specifications, including the layer names, input tensor, output tensor, and parameters. To properly evaluate our proposed model, we first developed a simple CNN model and used it as a baseline model for analyzing the effectiveness of other CNN models with attention modules. The second and the third models were built using two consecutive FLBs, TAM and FAM, respectively, followed by FCN. These models were developed to verify the effectiveness of the TAM and FAM modules. We developed model-4 for the analysis of whether TAM and FAM features complement each other when combined or not. The primary purpose of the last model is to analyze and represent the effectiveness of the MAM module when it is placed in various parts of the CNN model. We investigated the importance of the MAM mechanism by placing it in different regions of the CNN model. As shown in Table 4, model-5 achieved the best recognition score for all the classification tasks. Despite model-2, model-3, and model-4 results being lower than those of model-5, they remained higher than model-1. This indicates that our designed MAM mechanism demonstrated its capability to increase the CNN model performance by capturing suitable special and temporal features of the input speech spectrogram.

**Figure 7:**
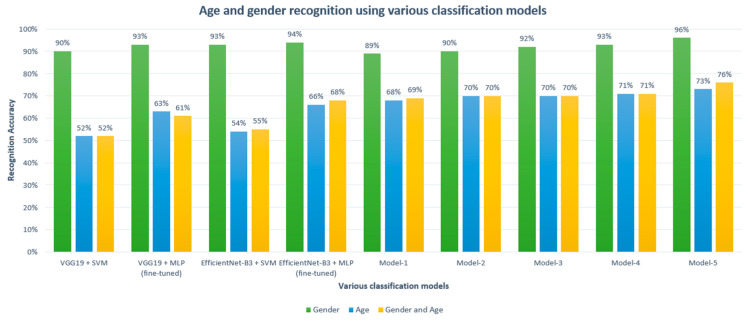
The recognition accuracy of various classification models for the classification of age, gender, and age-gender for the Common Voice dataset.

Moreover, we conducted experiments using transfer learning methodology to compare the performance and classification accuracy of our proposed model over other methods. VGG19 [51] and EfficientNet [37] image classification models were used to extract features from the input speech spectrogram, and an SVM [52] was used for classification. We extracted 4096-dimensional feature vectors for each audio file from the second fully connected layer of VGG19 by giving a speech spectrogram as an input image to the model. In the case of EfficientNet, we extracted 1536-dimensional feature vectors for each audio file from the global average layer of EfficientNet-B3 by giving speech spectrogram as an input image to the model. The extracted feature was used to train the SVM classifier with radial basis function (RBF) kernel and regularization parameter of one. Additionally, pre-trained models were fine-tuned for the gender, age, and age-gender classification tasks by replacing top fully connected layers of pre-trained models with our multi-layer perceptron network, which has two fully connected layers with 128 and 64 hidden neurons, respectively. The main reason for choosing VGG19 and EfficientNet pre-trained models for comparison with our proposed model is the similarity of input data to the model. The pre-trained models are very good at extracting useful features from the input image. As our model also uses speech spectrogram as input data, extract useful features, and perform classification task as pre-trained models do, we chose these models as baselines for comparing our proposed model. The same test set was used to evaluate the performance of the trained models. Figure 7 illustrates the accuracy score of various classification models for age, gender, and age-gender recognition obtained using the Common Voice dataset. The gender recognition results were above 90% for all the classification methods, except model-1. However, the age classification and age-gender classification results of pre-trained models were noticeably lower than those of all proposed models. Our proposed CNN model with the MAM mechanism achieved the best results in all three classification problems among the various classification models.

## 6. Conclusions and Future Direction

In this study, a novel CNN model with a MAM mechanism was proposed for the classification of age, gender, and age-gender using speech spectrograms. We addressed the two main issues in the field of age and gender recognition from speech signals. The first issue is the lack of the proper extraction of essential features, while the second issue is the design of an appropriate classification model. We designed a unique MAM mechanism to efficiently manage special and temporal salient features from the input data. Our proposed MAM uses a rectangular shape filter as a kernel in convolution layers and consists of two separate time and frequency attention mechanisms. The time-attention branch learns to detect the temporal cues of the input data. In contrast, the frequency attention module extracts the most relevant features to the target by focusing on the spatial features on the frequency axes. Both extracted features were then combined to complement one another and build more robust features for processing in the subsequent layers. We created an FLB composed of convolution, pooling, and batch normalization layers to extract local and global high-level features. We achieved the best recognition score for age and gender classification using the proper combination of FLB, MAM, and the FCN with the SoftMax classifier. We evaluated the performance and robustness of our proposed model over the Common Voice and Korean speech recognition datasets. We trained and tested our proposed model for age, gender, and age-gender classification problems. Additionally, we conducted experiments using the transfer learning method to evaluate the superiority of our proposed model. Our model achieved average accuracy scores of 96%, 73%, and 76% for the classification of gender, age, and age-gender tasks, respectively, for the Common Voice dataset. A 97% recognition rate was obtained for the gender and age classification using the Korean speech recognition dataset. For the age-gender recognition, the highest result obtained was 90% compared to the other results.

Even though our proposed model achieved the highest classification accuracy compared to the other models, there is still confusion between teens and twenties age groups in the Common Voice dataset. The prediction accuracy of teens age class was lower than 50% and mostly confused with twenties age class around 40%. It still requires conducting more research to decrease the confusion between teens and twenties age groups. Moreover, it is difficult to directly compare the results of the Common Voice dataset and Korean speech recognition dataset because of their age group labels difference. There is a significant difference between the classification scores of the Common Voice and the Korean speech recognition datasets. The reason behind this is the nature of the data labels in each dataset. Additionally, male recognition score is high compared to female recognition score in gender recognition task for both datasets because of data imbalance problem. As a result, it affects model generalizability.

We intend to apply this type of attention mechanism in the future for ASR, SER, and speech processing tasks. In the future, we will endorse and advance the architecture and compare it with a state-of-the-art established model to prove its significance. Additionally, testing our proposed model over other datasets would be advisable to evaluate the performance of the system. Our proposed MAM mechanism can be easily adapted to other CNN models to design an efficient and robust CNN model.

## Figures and Tables

**Table 1:** Detailed configuration specifications of our proposed model, including layer names, input tensor, output tensor, and parameters.

Layer Names	Input Tensor	Output Tensor	Kernel Size	Stride	Activation	Parameters
FLB						
Conv2D_1	64 × 64 × 3	32 × 32 × 120	9 × 9	2 × 2	ReLU	29,280
Batch_normalization_1	32 × 32 × 120	32 × 32 × 120	-	-	-	480
Max_pooling2D_1	32 × 32 × 120	16 × 16 × 120	2 × 2	2 × 2	-	0
Conv2D_2	16 × 16 × 120	16 × 16 × 256	5 × 5	1 × 1	ReLU	768,256
Max_pooling2D_2	16 × 16 × 256	8 × 8 × 256	2 × 2	1 × 1	-	0
Conv2D_3	8 × 8 × 256	8 × 8 × 384	3 × 3	1 × 1	ReLU	885,120
Batch_normalization_2	8 × 8 × 384	8 × 8 × 384	-	-	-	1536
MAM						
Conv2D_4	8 × 8 × 384	8 × 8 × 64	1 × 9	1 × 1	ReLU	221,248
Conv2D_5, Conv2D_6	8 × 8 × 64	8 × 8 × 64	1 × 3	1 × 1	ReLU	12,352
Batch_normalization_3	8 × 8 × 64	8 × 8 × 64	-	-	-	256
Conv2D_7	8 × 8 × 384	8 × 8 × 64	9 × 1	1 × 1	ReLU	221,248
Conv2D_8, Conv2D_9	8 × 8 × 64	8 × 8 × 64	3 × 1	1 × 1	ReLU	12,352
Batch_normalization_4	8 × 8 × 64	8 × 8 × 64	-	-	-	256
Concatenate_1	8 × 8 × 64	8 × 8 × 128	-	-	-	0
Batch_normalization_5	8 × 8 × 128	8 × 8 × 128	-	-	-	512
Concatenate_2	8 × 8 × 128	8 × 8 × 512	-	-	-	0
FCN						
Flatten	1 × 1 × 384	384	-	-	-	0
Dense_1	384	80	-	-	ReLU	30,800
Batch_normalization_7	80	80	-	-	-	320
Dense_2	80	12	-	-	SoftMax	972
Total parameters = 8,841,844						
Trainable parameters = 8,839,156						

**Table 2:** A detailed description of the utilized English subset of the Common Voice dataset.

Age Groups	Age Range (Years)	Train		Development		Test	
		Male	Female	Male	Female	Male	Female
Teens	<19	4249	1060	84	28	82	34
Twenties	19–29	18,494	3955	389	87	376	80
Thirties	30–39	13,662	4560	256	88	295	92
Forties	40–49	8684	2187	180	60	184	47
Fifties	50–59	4946	4467	116	87	116	89
Sixties	60–69	2835	1722	55	40	43	44
Total		52,870	17,951	1080	390	1096	386
		70,821		1470		1482	

**Table 3:** Detailed descriptions regarding age groups, age range, and utterances of the Korean speech recognition dataset.

Age Groups	Age Range (Years)	Train		Development		Test	
		Male	Female	Male	Female	Male	Female
Children	3~10	3341	3139	716	673	716	673
Adults	20~59	3971	2312	818	528	876	472
Elderly	60~79	3565	3109	764	666	764	667
Total		10,877	8560	2298	1867	2356	812
		19,437		4165		4168	

**Table 4:** Definition of the terms used to calculate evaluation metrics.

Actual Label	Predicted Label	Metrics Definition
Positive	Positive	True positive (TP)
Positive	Negative	False negative (FN)
Negative	Positive	False positive (FP)
Negative	Negative	True negative (TN)

**Table 5:** Classification accuracy (%) of the five different CNN models on the development set. All models were trained and evaluated for gender, age, and age-gender classification using the Common Voice and Korean speech recognition datasets. Numbers with bold fonts indicate the highest classification accuracy.

Input Feature	Model Type	Model Architecture	Common Voice			Korean Speech Recognition		
			Gender	Age	Age-Gender	Gender	Age	Age-Gender
**Speech Spectrogram**	Model-1	FLB + FLB + FCN	89	68	69	94	85	82
	Model-2	FLB + FLB + TAM + FCN	90	70	70	94	87	83
	Model-3	FLB + FLB + FAM + FCN	92	70	70	95	91	85
	Model-4	FLB + FLB + MAM + FCN	93	71	71	95	93	85
	Model-5	FLB + MAM + FLB+FCN	**96**	**73**	**76**	**97**	**97**	**90**

**Table 6:** A classification report of the proposed age and gender classification model on each dataset is provided to demonstrate precision, recall, F1-score, and weighted and unweighted accuracy results. F and M denote the female and male genders, respectively. Numbers with bold fonts indicate the average classification accuracy.

Common Voice Analysis					Korean Speech Recognition Analysis				
Class Category	Precision	Recall	F1-Score	Utterances	Class Category	Precision	Recall	F1-Score	Utterances
F-teens	0.78	0.53	0.63	34	F-children	0.75	0.97	0.85	673
F-twenties	0.72	0.8	0.76	80	F-adult	0.99	0.88	0.93	472
F-thirties	0.8	0.79	0.8	92	F-elderly	0.95	0.95	0.95	667
F-forties	0.76	0.68	0.72	47	M-children	0.91	0.75	0.82	716
F-fifties	0.8	0.85	0.83	89	M-adult	0.99	0.83	0.91	876
F-sixties	0.87	0.89	0.88	44	M-elderly	0.86	0.99	0.92	764
M-teens	0.68	0.52	0.59	82					
M-twenties	0.72	0.81	0.76	376					
M-thirties	0.75	0.73	0.74	295					
M-forties	0.76	0.72	0.74	184					
M-fifties	0.79	0.75	0.77	116					
M-sixties	0.89	0.77	0.82	43					
Weighted Accuracy	0.76	0.75	0.75	1482		0.91	0.9	0.9	4168
Unweighted Accuracy	0.78	0.74	0.75			0.91	0.9	0.9	
**Accuracy**	**0.75**					**0.9**			

**Table 7:** Classification of the proposed age classification model on each dataset presents the precision, recall, F1-score, and weighted and unweighted accuracy results. Numbers with bold fonts indicate the average classification accuracy.

Common Voice Analysis					Korean Speech Recognition Analysis				
Class Category	Precision	Recall	F1-Score	Utterances	Class Category	Precision	Recall	F1-Score	Utterances
Teens	0.78	0.47	0.58	116	Children	0.96	0.97	0.97	1389
Twenties	0.66	0.83	0.73	456	Adult	0.98	0.92	0.95	1348
Thirties	0.76	0.65	0.7	387	Elderly	0.95	0.99	0.97	1431
Forties	0.72	0.69	0.7	231					
Fifties	0.87	0.77	0.81	205					
Sixties	0.7	0.82	0.76	87					
Weighted Accuracy	0.73	0.72	0.72	1482		0.96	0.96	0.96	4168
Unweighted Accuracy	0.75	0.7	0.71			0.96	0.96	0.96	
**Accuracy**	**0.72**					**0.96**			

**Table 8:** Classification report of the proposed gender classification model for each dataset presents the precision, recall, F1-score, and weighted and unweighted accuracy results. Numbers with bold fonts indicate the average classification accuracy.

Common Voice Analysis					Korean Speech Recognition Analysis				
Class Category	Precision	Recall	F1-Score	Utterances	Class Category	Precision	Recall	F1-Score	Utterances
Male	0.97	0.97	0.97	1096	Male	0.96	0.97	0.96	2356
Female	0.92	0.91	0.92	386	Female	0.96	0.95	0.95	1812
Weighted Accuracy	0.96	0.96	0.96	1482	Weighted Accuracy	0.96	0.96	0.96	4168
Unweighted Accuracy	0.94	0.94	0.94		Unweighted Accuracy	0.96	0.96	0.96	
**Accuracy**	**0.96**					**0.96**			

## Data Availability

Not applicable.
